# A Closer Look at the NS1 of Influenza Virus

**DOI:** 10.3390/v1031057

**Published:** 2009-11-26

**Authors:** William G. Dundon, Ilaria Capua

**Affiliations:** OIE/FAO and National Reference Laboratory for Avian influenza and Newcastle Disease, Istituto Zooprofilattico Sperimentale delle Venezie, Viale dell’ Università, 10, Legnaro (PD), 35020, Italy; E-Mail: icapua@izsvenezie.it

**Keywords:** Non-Structural 1 protein, carboxy terminal, avian influenza, vaccination, DIVA

## Abstract

The Non-Structural 1 (NS1) protein is a multifactorial protein of type A influenza viruses that plays an important role in the virulence of the virus. A large amount of what we know about this protein has been obtained from studies using human influenza isolates and, consequently, the human NS1 protein. The current global interest in avian influenza, however, has highlighted a number of sequence and functional differences between the human and avian NS1. This review discusses these differences in addition to describing potential uses of NS1 in the management and control of avian influenza outbreaks.

## 1. Introduction

The NS1 protein of influenza A viruses has been attracting a lot of attention recently given its multifunctional role and association with the virulence of the virus. The long list of functional characteristics and interactors of NS1 have been reviewed extensively by Krug *et al.* [1], Lin *et al.* [2] and more recently by Hale *et al*. [3] and are not the scope of this review. These authors have concentrated primarily on the NS1 from human isolates since, prior to the 1997 Hong Kong H5N1 outbreak, there was very little information available on the NS1 of avian influenza isolates. This has changed, dramatically over the last decade and now, thanks to several influenza genome sequencing projects the influenza scientific community is in a situation whereby the genetic characteristics of influenza A from different species can be compared [4–6]. Such comparisons between human and avian influenza viruses highlight a number of important and noteworthy differences that are discussed below.

The NS1 protein has also been investigated by various groups to determine its possible use in AI vaccine development. In addition, the protein has been studied as a possible candidate for use in so-called DIVA (Differentiation of Infected from Vaccinated Animals) strategies in the control of AI. Both approaches will be discussed.

## 2. Comparison between the NS1 of human and avian influenza viruses

### 2.1. Structural differences

The NS1 protein can be separated into distinct domains. The first 73 amino acids (aa) of the protein form a double-stranded RNA binding domain that counteracts the host immune response by blocking the synthesis of type I interferon. The carboxy terminal effector domain interacts with a number of cellular proteins and contains both nuclear export and import sequences along with a recently described nucleolar localization sequence [1–3]. The C-terminal domain has also been implicated in the enhancement of NS1’s interferon antagonist properties by stabilizing its dimeric structure, a prerequisite for RNA binding [7,8]

Several groups have reported the resolution of the three dimensional structure of either the RNA binding or the effector domain of NS1 [9–12] but it was Bornholdt and Prasad [13] who finally managed to resolve the complete structure at the end of 2008 by introducing two mutations (R38A and K41A) into the recombinant NS1 that prevented aggregation of the protein at the protein concentrations required for crystallographic studies. The report by Bornholdt and Prasad [13] provided the influenza community with possible explanations to several unanswered questions regarding NS1. In brief, their crystallographic analysis revealed that the NS1 molecules associate with each other to form a tubular structure that allows the protein to sequester double stranded RNA of varying lengths while at the same time interacting with other host proteins through binding sites on the outer surface. This explains how the protein can simultaneously perform the many tasks ascribed to it.

Prior to the resolution of the full NS1 structure, a comparison of the X-ray crystallographic structure of the effector domain of an avian NS1 from A/Duck/Albany/76 was undertaken by Hale *et al.* [8]. The resolved structure was compared to the effector domain structure of a human influenza virus A/Puerto Rico/8/34 previously resolved by Bornholdt and Prasad [14] and a number of differences were identified. These included a difference in the orientation of a β-hairpin between the viruses. This β-hairpin is also referred to as the 140-loop and as yet no biological function has been associated to it although residues close to the loop have been implicated in the nuclear export of NS1 into the cytoplasm of the host cell [15]. A second orientation difference between the two viruses was also identified at residue Tyr-89 which is involved in the NS1-mediated binding of phosphoinositide 3-kinase (P13K) [16] although no functional difference was observed [10,16]. Finally, Trp-187 implicated in the binding of the 30kDA subunit of the cleavage and polyadenylation factor (CPSF30) also adopted a different orientation between the two structures [17]. Interestingly, with reference to Trp-187, previous work has shown that the CPSF30-binding site on the NS1 of A/Duck/Albany/76 is functional while the human isolate A/Puerto Rico/8/34 does not interact with CPSF30 [18,19]. Nevertheless and despite these interesting findings, A/Puerto Rico/8/34 is a laboratory strain which may not completely represent other wild-type human viruses so care must be taken in drawing conclusions until the resolution of further NS1 structures from both avian and human viruses are undertaken and then compared at both a functional and structural level.

## 2.2. Sequence differences

Based on their amino acid sequence the NS1 of influenza A viruses can be divided into two groups refereed to as alleles, a term coined in 1989 by Treanor *et al.* [20]. The homology between the two alleles can be as low as 62%. The allele B group is made up exclusively of NS1 proteins from avian influenza viruses while allele A consists of proteins from both avian and mammalian sources. Studies have shown that allele A viruses have a replicative advantage in mammalian hosts and that they are under continual selection pressure as opposed to allele B viruses [21]. There are no reports of functional differences associated with the different alleles to date. The structural differences described above between human and avian NS1s may be due to the fact that the NS1 of A/Duck/Albany/76 is from allele B. Therefore the future resolution of the complete structure of an allele A NS1 would be of particular interest.

### 2.3. Deletions

The highly pathogenic avian influenza (HPAI) viruses of the H5N1 subtype have emerged as a devastating pathogen of poultry in Asia, Europe and Africa. The NS1 protein of this highly pathogenic virus has been shown to posses a unique 5 aa deletion (15 nucleotides) at position 80 to 84 resulting in a protein that is 5 aa shorter than the normal 230 for avian influenza NS1s. An analysis of the public sequence databases reveals that this deletion emerged in H5N1 isolates in 2000 and has persisted ever since outlasting H5N1 viruses with a 230 aa NS1 protein. Indeed, the last H5N1 NS1 without the 5 aa deletion was isolated in Kazakhstan in 2006 (Genbank ACJ53834). This would suggest that the deletion confers an, as yet, unidentified advantage on the viruses that possess them.

The H5N1 virus (A/Viet Nam/1203/2004) used by Bornholdt and Prasad to resolve the 3D structure of NS1 possesses the 5 aa deletion [13]. The deletion is located in the linker region between the N-terminal RNA-binding domain and the C-terminal effector domain of the protein and so Bornholdt and Prasad suggest that it may allow for modulation of NS1’s affinity for dsRNA by influencing the positioning of the RNA-binding domain dimers. The deletion could also explain the observed conformational changes observed between the RNA-binding domain of the H5N1 virus and those of the H1N1 and H3N1 which do not possess the 5 aa deletion [13].

Long *et al.* [22] have attempted to associate a biological significance to this deletion by generating recombinant viruses with and without the 15 nt deletion and experimentally infecting both chickens and mice. Their results demonstrated that in association with a D92E shift, previously shown to confer resistance to antiviral cytokines in a pig model [23], that the 15 nt deletion contributed to the virulence of the recombinant viruses in both animal models. However, since the increased virulence observed was always associated with the D92E shift and could not be conferred on a virus with an Asparagine at position 92, there is debate as to whether the observations reported were due to the 15 nt deletion or not [3].

### 2.4. Binding domains

A large scale sequence analysis of avian influenza viruses revealed that the c-terminal four residues of the NS1 protein is a potential PDZ domain ligand [24]. PDZ domains are recognition modules that organize a variety of cell-signaling assemblies and it has been speculated that binding of these domains by NS1 might interfere with host cell signaling [25]. It was clearly shown by Obenauer *et al.* [24] that avian-like NS1 c-terminal residues (ESEV or EPEV) bind human PDZ domain-containing proteins while human c-terminal residues do not. A second study by Jackson *et al.* [26] employed reverse genetics to demonstrate that avian-like c-terminal residues modulate pathogenicity in a mouse model.

The majority of avian influenza viruses have been shown to possess a glutamic acid (E) at amino 227 of the NS1 protein compared to human isolates which contain an arginine (R) [27]. Finkelstein *et al.* [28] observed that the H1N1 that caused 1918 “Spanish flu” pandemic had a K at amino acid 227 and more recently this lysine residue has also been identified in H5N1 AI viruses isolated from the Kingdom of Saudi Arabia and H9N2 viruses from Israel [29] (Genbank). This mutation is therefore being considered by some groups as an important marker of viruses that may pose a public health threat.

It has been reported by Heikkinen *et al.* [30] that class II SH3-binding motifs are commonly found among avian NS1 (residues 212 to 217) but rarely among human isolates. Of the four human isolates that possess this motif, three were due to zoonotic transmissions to humans [30]. SH3 domains are small protein modules that mediate protein-protein interactions and are often found in proteins that regulate cellular signalling pathways, cytoskeletal organization and membrane trafficking. It is hypothesized that exploiting the cellular signalling machinery of the host might provide viruses that possess these SH3-binding motifs with increased replicative ability and/or pathogenicity.

### 2.5. Carboxy terminal truncations and elongations

As mentioned previously the c-terminal of the NS1 protein has been implicated in the enhancement of the protein’s interferon antagonist properties by stabilizing its dimeric structure [7,8]. The c-terminal also possesses an effector domain that can inhibit 3′-end processing of cellular pre-mRNA by interaction with the 30kDA subunit of the cleavage and polyadenylation factor (CPSF) and can prevent transport of cellular mRNA to the cytoplasm by interaction with the poly(A)-binding protein II (PABII) [17,31]. In addition, the c-terminal has been shown to have one of two nuclear location signals (NLS) that play an important role in the entry and accumulation of the protein in the nuclei of infected cells [32,33]. More recently, Melén *et al*. [33] located the precise residues that make up the NLS2 and have also identified a nucleolar localization signal (NoLS) sequence that shares residues with NLS2.

Studies of the NS1 protein of H7N1 and H7N3 avian influenza isolates from Italy and Pakistan have revealed truncations of the carboxy terminal of the protein following extensive circulation in poultry [34,35]. In order to determine whether these truncations were confined just to AI viruses of the H7 subtype we have carried out a detailed analysis of 7,141 NS1 proteins sequences from influenza A viruses of avian, equine, human, and swine origin deposited in the public databases and accessible through the Influenza Virus Resource (http://www.ncbi.nlm.nih.gov/genomes/FLU/FLU.html) (up to August 2009).

**Figure 1.**
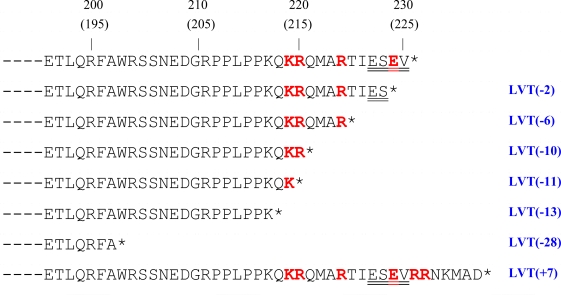
Schematic representation of the length variation types (LVTs) identified in the NS1 protein. The consensus sequence of avian influenza NS1 proteins are shown. Amino acids from the NLS2/NoLS [32,33] are shown in bold red while the PL motif [24] is doubly underlined.

Overall, seven length variation types (LVTs) from the wild-type length of 225/230 aa can be identified. These LVTs consist of a 2 [LVT(−2)], 6 [LVT(−6)], 10 [LVT(−10)], 11 [LVT(−11)], 13 aa [(LVT(−13)] or 28 aa [(LVT(−28)] c-terminal truncation or a 7 aa [LVT(+7)] elongation resulting in proteins of 223 aa (for H5N1 viruses only), 224 aa, 220 aa (215 aa for some H5N1 viruses), 219 aa, 217 aa (212 aa for H5N1 viruses), 202 aa and 237 aa (232 aa for H5N1 viruses) respectively (Table 1 and Figure 1).

Analysis of the nucleotide sequence reveals that all the truncated proteins are the result of a single base change resulting in the creation of a stop codon except for the 6 aa truncation which is the result of two nucleotide changes in the same codon. The 7 aa elongations are due to the creation of an amino acid from a stop codon by a single nucleotide change.

The LVT(−2) was identified in avian H5N1 viruses only. Specifically, the 2 aa truncation was seen among a group of 72 H5N1 AI viruses isolated between 2004 to 2006 in Vietnam, Hong Kong and China [36,37]. Although the authors undertook a detailed phylogenetic analysis of these viruses no comment was made on the length variation of the NS1 proteins. It is of particular interest as the truncation involves the PL (PDZ ligand) motif described by Obenauer *et al.* [24]. As mentioned above, Jackson *et al.* [26] investigated the effect of changes to the PL motif in the mouse model using reverse genetics by creating several recombinant viruses, one of which completely lacked the 4 c-terminal amino acids of the PL motif. This recombinant viruses displayed an attenuated phenotype when compared to recombinant viruses with an intact PL motif when tested for viral growth kinetics and in a mouse infection model. It would therefore be expected that the H5N1 viruses with a LVT(−2) would also behave differently from viruses with an intact PL motif and this observation merits further investigation.

The LVT(−6) is also only found in one group of viruses, namely, the avian influenza H7N1 viruses isolated from domestic poultry in Northern Italy between 1999 and 2001 [34]. The 6 aa truncation is due to a mutation of a threonine at position 225 to a stop codon. This mutation involves changing two nucleotides within the same codon and this may explain why LVT(−6) has not been identified in other influenza A viruses given that a double mutation is a much rarer event. Of note is that, compared to the H7N1 isolates with a non-truncated protein which were all of low pathogenicity, all of the viruses with a LVT(−6) were highly pathogenic and caused severe disease in domestic poultry. Whether the truncated NS1 plays a role in this increased pathogenicity is presently unknown.

The LVT(−10) was identified in human (H2N2, H3N2 and H5N1) and avian (H7N1 and H9N2) but not in equine and swine influenza A viruses. The presence of the 10 aa truncation in the human H2N2 and H3N2 is confined to the 5 year period between 1967 and 1972 and has not been seen since [38]. The two human H5N1 viruses of avian origin are of particular interest. This truncation completely removes the PL motif described by Obenauer *et al.* [24]. One of these H5N1 viruses (A/Viet Nam/1203/2004) has previously been shown by Govorkova *et al.* [39] to be particularly virulent in the ferret model suggesting that, although the avian like PL motif seems to play a role in the pathogenesis of H5N1 viruses, it does not appear to be a prerequisite for virulence [26]. Additional analysis of virus A/Viet Nam/1203/2004 using reverse genetics showed that by replacing the 220 aa NS1 protein with a full-length 225 aa NS1 protein the virus was significantly attenuated in a ferret model suggesting an important role for this 10 aa truncation in the pathogenesis of H5N1 viruses [40].

A small number of H9N2 avian influenza isolates also possess the LVT(−10). These viruses are part of a group of seven homogeneous isolates from chicken (n=4) and quail (n=3) obtained in the United Arab Emirates and studied by Amir *et al.* [41]. Interestingly, the quail isolates, which did not produce any overt clinical signs in the field, all had a full-length 230 aa long NS1. In contrast, the chicken isolates were characterised by general congestion, severe tracheitis and acute onset mortality and three out of four of them had an NS1 with LVT(−10). In challenge experiments it was also shown by Amir *et al.* [41] that there was a clear biological difference between the quail and chicken isolates; the quail isolates replicated in chickens but failed to transmit to contact birds while the chicken isolates both replicated and transmitted to contact birds. It is possible that NS1 plays a role in the transmission of the virus and so the non-chicken adapted strain had more difficulty transferring to chickens. Although the sample group is small, it is tempting to think that the results of Amir *et al.* [41], with specific reference to the NS1, is also evidence for host adaptation from quail to chicken.

LVT(−11) is found predominantly in swine origin viruses; that is the classical swine influenzas of subtypes H1N1, H1N2 and H3N2 and the presently circulating human pandemic H1N1 virus. There are, however, a small number (n=24) of avian influenza viruses, one of which is of swine origin (e.g., A/turkey/Chile/28317-6504-3/2009) and a slightly larger number (n=40) of equine H3N8 that posses an NS1 of 119 aa. Interestingly, a further analysis of the NS1 nt sequences from these viruses reveals that there is no phylogenetic relationship between the NS1 originating from swine and those from horses indicating that these truncations are not due to random mutational events. The characteristics of NS1 proteins with a LVT(−11) would be expected to be very similar to those possessing a LVT(−10) but to date there is no biological data available for this group of viruses. Given the interest in pandemic H1N1 a further characterization of the truncated NS1 would be opportune.

The LVT(−13) is the most common of the variations in the NS1 protein with seven avian influenza subtypes possessing it. The majority of the H9N2 (69%) subtype viruses analysed possess the 13 aa truncation. Of the avian H6N1 viruses that possess the LVT(−13), all were isolated in China or Hong Kong from minor poultry species (e.g. quail, chukar, guinea fowl, partridges and pheasants) from 2000 to 2005 [42]. Of interest is that in this study only one H6N1 AI virus was isolated from 8,788 chicken specimens examined suggesting that the H6N1 viruses with a LVT(−13) truncated proteins can infect minor poultry species more easily than chickens.

For the H7N3 viruses with the LVT(−13) all except one were isolated in Pakistan and Afghanistan between 1995 and 2005 [35]. In this study we also identified two Pakistani H7N3 viruses with a 230 aa NS1 that were genetically distinct from the viruses with a truncated protein indicating that within the H7N3 Pakistan virus populations there were at least three viral subpopulations that circulated in the 10 year period investigated. All of the viruses were highly pathogenic causing severe disease in poultry [43]. The LVT(−13) removes a significant number (n=4) of residues from the NLS [32,33] in addition to the binding site of PABII [31]. Despite this, it is the most common LVT found among influenza A viruses clearly questioning the significance of PABII binding and the NLS in the pathogenesis and transmission of these viruses.

LVT (−28) was only observed in avian (n=5) and human H1N1 (n=3) isolates. This is the largest of the truncation removing over 12% of the protein and was first described in the two human isolates by Kyrstal *et al.* [44]. The removal of such a significant stretch of the c-terminal of the protein would be expected to have an effect on the stability of the dimeric structure which in turn would alter the interferon antagonist properties of the NS1 as described by Wang *et al.* [8]. It is possible that given the low number (n=8) of isolates identified with this 28 aa truncation that this LVT is a laboratory artefact due to passaging in cells or embryonated eggs. It has already been reported that large c-terminal deletions can be tolerated by influenza A when grown in eggs or cell culture [45]. It would be of interest to investigate the ability of the NS1 proteins from these virus to dimerize and abrogate IFN production in infected cells.

Only eight AI viruses have the 7 aa elongation which is predominant among human H1N1 and H2N2 isolates. These AI viruses are a wild bird isolate of the H13N2 subtype, a chicken H7N3 virus isolated in our laboratory from Northern Italy in 2007 and six H5N1 viruses originating from Vietnam and China in 2003 and Nigeria in 2006. A more detailed sequence analysis of the PL motif of each of the virus reveals that the Vietnamese, Italian and wild bird isolates all have the avian-like ESEV motif followed by the 7 aa extension. Jackson *et al.* [26] created a recombinant virus with an avian-like ESEV motif followed by a 7 aa extension. This recombinant viruses, however, proved to be attenuated in both growth kinetics and in infection of mice. These results therefore suggest that avian viruses with LVT(+7) elongated NS1 proteins may not replicate in other species as readily as viruses with shorter NS1 proteins. Indeed, given the large number of human isolates with LVT(+7) the host would appear to be important in determining whether the NS1 protein is elongated or not.

An analysis of the NS1 sequence data available has clearly shown that the length variations seen between influenza A viruses isolated from different species in not a random event. Given the fact that it is not a random event, the removal or addition of specific amino acid residues at the c-terminal of the protein must in some way confer an advantage or disadvantage on the viruses. In some of the truncations seen the PL, NLS and the PABII binding sites are directly effected but it is not clear at present to what extent this alters pathogenicity or transmission of the viruses that posses them. Indeed there is conflicting evidence that suggests that the PL is probably not an essential virulence factor for influenza A viruses. In this review we have listed several examples of highly pathogenic H7 and H5 viruses that have truncations that remove the PL but still maintain their pathogenicicty. Further work on the PL domain is therefore required to resolve this question.

## 3. NS1 and vaccination

Highly pathogenic avian influenza (HPAI) is a devastating disease that has caused enormous economic losses in the poultry industry worldwide. International organisations have issued a series of recommendations aimed at controlling AI [46]. In addition to direct control measures based on biosecurity, restriction policies and stamping out, the appropriate use of vaccines is encouraged to maximise eradication efforts. Historically the use of vaccination against AI has been discouraged or banned as it was feared that it would prevent the rapid diagnosis of the disease by hiding clinical manifestations in poultry.

A number of different types of vaccination strategies for AI are now available [46]. These range from conventional inactivated vaccine using low pathogenicity avian influenza (LPAI) virus with homologous HA to the field virus, to genetically engineered or reassortant viruses to DNA vaccination [46].

It is known that blocking or reducing the function of the NS1 can attenuate the influenza virus by allowing the host to generate a strong interferon response against the virus [1–3]. Attenuated viruses are ideal candidates for live-attenuated vaccines which have the added advantages over inactivated vaccines of triggering mucosal immune responses and inducing cell mediated immunity in the host. For this reason a number of different groups have been focusing their attention on the development of vaccines based on the genetic manipulation of the *ns1* gene

Wang *et al*. [47] recently investigated the potential use of a series of reassortant viruses that had various deletions in their *ns1* genes. By passaging a well characterised virus (A/Turkey/Oregon/71) with a previously identified 10 nucleotide deletion in the *ns1* gene they were able to clone and identify more that 20 *ns1* genes of different sizes. All of these *ns1* genes variants were selected and nine reassortant viruses were created and characterised further. The reassortants were tested for their protective ability in SPF chickens following challenge with a heterologous virus of the same HA subtype and it was clearly shown that two of the reassortant viruses could be used as potential live attenuated vaccines in poultry.

Zhirnov *et al.* [48] have determined the protective efficacy of DNA vaccination in poultry using a combination of conserved proteins namely the matrix (M1) and nucleoprotein (NP). However, the authors observed that by including an NS1-encoding plasmid as part of the M1/NP immunization protocol they could significantly improve protection to virus challenge in mice and chickens. Although not confirmed by the authors, this effect is most likely due to the induction of cellular immunity to the NS1, a response which has previously been reported in human peripheral blood mononuclear cells [49].

Similar work has been carried out by Steel *et al*. [50]. In this report the authors created a set of experimental live-attenuated vaccines based on a recombinant H5N1 virus (A/Viet Nam/1203/04) with a glutamic acid at the residues 627 of the PB2 and one of a series of truncated NS1 proteins. A recombinant vaccine with a 99 aa long NS1 protein completely protected chickens from lethal challenge from both a homologous (A/Viet Nam/1203/04) and heterologous (A/egret/Egypt/01/06) H5N1 virus confirming the potential of such vaccines in the control of avian influenza

## 4. NS1 DIVA

It is known that vaccination prevents clinical disease, increases resistance to infection and reduces virus shedding levels, but does not prevent infection if birds are challenged with a sufficiently high dose of virus [51,52]. For this reason, vaccinated birds may still become infected and shed virus into the environment without displaying any clinical signs, and therefore they represent a means of spreading infection.

In order, therefore, to support eradication efforts of AI infections in poultry, the implementation of “DIVA” vaccination strategies, enabling the Differentiation of Infected from Vaccinated Animals have been recommended by international organizations like the OIE (World Organization for Animal Health) and FAO (United Nations Food and Agricultural Organization). These systems allow for the detection of field exposure in vaccinated flocks and through this, infected flocks may be properly managed, thus interrupting the perpetuation of the infectious cycle.

Several “DIVA” systems have been developed to date although they have some limitations in the field [53–57]. A promising system, based on the detection of antibodies against the NS1 of AI has been deemed a good candidate [58–59]. The NS1 protein is synthesized in large amounts in infected cells but is not incorporated into the mature virions, and for this reason represents the ideal candidate to elicit a specific immune response only in the presence of active viral replication.

A paper by Birch-Machin *et al.* [60] reported that antibodies to NS1 could be detected in serum samples of ponies experimentally infected with equine influenza virus, but not in animals vaccinated with whole inactivated virus. Likewise a study by Ozaki *et al.* [61] identified antibodies to the NS1 exclusively in the sera of mice infected with equine influenza viruses and not in those mice immunized with inactivated virus.

A number of groups have investigated the feasibility of a NS1 DIVA for AI. Tumpey *et al*. [58] experimentally infected poultry and evaluated their ability to induce antibodies reactive to NS1 recombinant protein produced in *Escherichia coli* or to chemically synthesized NS1 peptides. Immune sera were obtained from chickens and turkeys inoculated with live AI virus, inactivated purified vaccines, or inactivated commercial vaccines. Seroconversion to positivity for antibodies to the NS1 protein was achieved in birds experimentally infected with multiple subtypes of influenza A virus, as determined by enzyme-linked immunosorbent assay (ELISA) and Western blot analysis. In contrast, animals inoculated with inactivated gradient-purified vaccines had no seroconversion to positivity for antibodies to the NS1 protein, and animals vaccinated with commercial vaccines had low, but detectable, levels of NS1 antibodies. The use of a second ELISA with diluted sera identified a diagnostic test that results in seropositivity for antibodies to the NS1 protein only in infected birds. For the field application phase of this study, serum samples were collected from vaccinated and infected poultry, diluted, and screened for anti-NS1 antibodies. Field sera from poultry that received commercial AI vaccines were found to possess antibodies against AI virus, as measured by the standard agar gel precipitin (AGP) test, but they were negative by the NS1 ELISA. Conversely, diluted field sera from AI-infected poultry were positive for both AGP and NS1 antibodies.

Another study by Zhao *et al.* [59] reported the development of a similar ELISA based DIVA strategy to differentiate AI-infected chickens versus chickens immunized with inactivated avian influenza virus. A recombinant nonstructural protein (NS1) cloned from an H9N2 AI virus was expressed and purified from *Escherichia coli* and used as the diagnostic antigen. Similar to the study by Tumpey *et al.* [58], antibodies to the NS1 protein were only detected in the sera of chickens experimentally infected with AI but not in the sera of chickens immunized with inactivated vaccine.

One of the limitation of the NS1 DIVA approach is the purity of the vaccines used. Commercial vaccines are normally only partially purified and so, although inactivated, they may contain residual NS1 protein which can, in turn, effect the performance and interpretation of an ELISA design to detect antibodies to NS1. Indeed, from the work of Zhao *et al.* [59] there is an indication that the vaccine they used in their study was unsuitable for the above reason. Likewise, these authors, by intravenously challenging their experimental birds with virus, failed to mimic field infection dynamics and possibly induced a stronger immune response to the NS1 protein.

Some work has been performed to determine the onset of the antibody response in chickens and turkeys to the NS1 protein. The data obtained indicate that the duration of the antibody response and the variability between species may detract from the performance and utility of an NS1 DIVA strategy [62,63]. So, despite the apparently promising results from the work by Tumpey *et al.* [58] and Zhao *et al.* [59], further work needs to be undertaken and full field validation under different circumstances is required before an NS1 DIVA strategy can be proposed to the veterinary community.

## 5. Conclusion

The NS1 is a fascinating protein with a surprisingly large number of functions ascribed to it for a relatively small protein. The resolution of the three dimensional structure has gone a long way in explaining the multifunctionality of the protein. Likewise, the huge increase in influenza A genomes that are being sequenced has allowed the influenza community to identify similarities and differences between viruses that infect birds and those that infect mammals. Despite this many questions remained unanswered particularly in relation to the observed deletions and truncations that are stably maintained by the viruses that possess them. An eventual explanation for these phenomena will no doubt add a further understanding of NS1s involvement in the biology of influenza A viruses.

Developments in vaccine strategies using the NS1 are ongoing and should provide good alternatives for the control of Avian influenza. They will, however, require complete field validation and the existence of validated companion kits before they become part of routine control strategies for AI.

## Figures and Tables

**Table 1.** Length variation types (LVTs) in the NS1 protein of influenza A viruses (August 2009).

NS1 length aa (LVT)														
Species	Subtype	202	212	215	217	219	220	223	224	225	230	232	237	Total
Avian	H1N1					6					71			77
Avian	H1N2					2					5			7
Avian	H3N2					14					49			63
Avian	H3N8				1						108			109
Avian	H4N6	1				1					101			103
Avian	H4N8										34		1	35
Avian	H5N1		2		8			72		1123	78	6		1289
Avian	H5N2				17						144			161
Avian	H5N9	2									4			6
Avian	N6N1	1			70						83			154
Avian	H6N2				42						79			121
Avian	H7N1	1					11		34		33			79
Avian	H7N2					1					156			157
Avian	H7N3				30						107		1	138
Avian	H9N2				393		3				171			567
Avian	H13N2										3		1	4
Human	H1N1	3			3	509*					720		104	1339
Human	H2N2				1		13				2		74	90
Human	H3N2					2	9				1825		167	2003
Human	H5N1			2						125	17			144
Human	H9N2				1						4			5
Swine	H1N1				5	109					53		3	170
Swine	H1N2				3	66					14			83
Swine	H2N3					2								2
Swine	H3N1					5					1			6
Swine	H3N2				6	34					48		11	99
Equine	H3N8				2	40					88			110

	Total	8	2	2	582	791	36	72	34	1248	3998	6	362	7141

* Swine origin pandemic H1N1

## References

[1] Krug RM, Yuan W, Noah DL, Latham AG (2003). Intracellular warfare between human influenza viruses and human cells: the role of the viral NS1 protein. Virology.

[2] Lin D, Lan J, Zhang Z (2007). Structure and function of the NS1 protein of influenza A virus. Acta Biochim Biophys Sin (Shanghai).

[3] Hale BG, Randall RE, Ortìn J, Jackson D (2008). The multifunctional NS1 protein of influenza A viruses. J Gen Virol.

[4] Salzberg SL, Kingsford C, Cattoli G, Spiro DJ, Janies DA, Aly MM, Brown IH, Couacy-Hymann E, De Mia GM, Dung do H, Guercio A, Joannis T, Maken Ali AS, Osmani A, Padalino I, Saad MD, Savić V, Sengamalay NA, Yingst S, Zaborsky J, Zorman-Rojs O, Ghedin E, Capua I (2007). Genome analysis linking recent European and African influenza (H5N1) viruses. Emerg Infect Dis.

[5] Bao Y, Bolotov P, Dernovoy D, Kiryutin B, Zaslavsky L, Tatusova T, Ostell J, Lipman D (2008). The Influenza Virus Resource at the National Center for Biotechnology Information. J Virol.

[6] Cattoli G, Monne I, Fusaro A, Joannis TM, Lombin LH, Aly MM, Arafa AS, Sturm-Ramirez KM, Couacy-Hymann E, Awuni JA, Batawui KB, Awoume KA, Aplogan GL, Sow A, Ngangnou AC, El Nasri Hamza IM, Gamatié D, Dauphin G, Domenech JM, Capua I (2009). Highly pathogenic avian influenza virus subtype H5N1 in Africa: a comprehensive phylogenetic analysis and molecular characterization of isolates. PLoS One.

[7] Wang W, Riedel K, Lynch P, Chien CY, Montelione GT, Krug RM (1999). RNA binding by the novel helical domain of the influenza virus NS1 protein requires its dimer structure and a small number of specific basic amino acids. RNA.

[8] Wang X, Basker CF, Williams BRG, Silverman RH, Palese P, García-Sastre A (2002). Functional replacement of the carboxy-terminal two-thirds of the influenza A virus NS1 protein with short heterologous dimerization domains. J Virol.

[9] Das K, Ma LC, Xiao R, Radvansky B, Aramini J, Zhao L, Marklund J, Kuo RL, Twu KY, Arnold E, Krug RM, Montelione GT (2008). Structural basis for suppression of a host antiviral response by influenza A virus. Proc Natl Acad Sci USA.

[10] Hale BJ, Barclay WS, Randall RE, Russell RJ (2008). Structure of an avian influenza A virus NS1 protein effector domain. Virol.

[11] Cheng A, Wong SM, Yuan YA (2009). Structural basis for dsRNA recognition by NS1 protein of influenza A virus. Cell Res.

[12] Xia S, Monzingo AF, Robertus JD (2009). Structure of NS1A effector domain from the influenza A/Udorn/72 virus. Acta Crystallogr D Biol Crystallogr.

[13] Bornholdt ZA, Prasad BV (2008). X-ray structure of NS1 from a highly pathogenic H5N1 influenza virus. Nature.

[14] Bornholdt ZA, Prasad BV (2006). X-ray structure of influenza virus NS1 effector domain. Nat Struct Mol Biol.

[15] Li Y, Yamkita Y, Krug RM (1998). Regulation of a nuclear export signal by an adjacent inhibitory sequence: the effector domain of the influenza virus NS1 protein. Proc Natl Acad Sci U S A.

[16] Hale BG, Jackson D, Chen YH, Lamb RA, Randall RE (2006). Influenza A virus NS1 protein binds p85beta and activates phosphatidylinositol-3-kinase signaling. Proc Natl Acad Sci U S A.

[17] Nemeroff ME, Barabino SML, Li Y, Keller W, Krug RM (1998). Influenza virus NS1 protein interacts with the cellular 30 kDa subunit of CPSF and inhibits 3′end formation of cellular pre-mRNAs. Molecular Cell.

[18] Hayman A, Comely S, Lackenby A, Hartgroves LC, Goodbourn S, McCauley JW, Barclay WS (2007). NS1 proteins of avian influenza A viruses can act as antagonists of the human alpha/beta interferon response. J Virol.

[19] Kochs G, García-Sastre A, Martínez-Sobrido L (2007). Multiple anti-interferon actions of the influenza A virus NS1 protein. J Virol.

[20] Treanor JJ, Snyder MH, London WT, Murphy BR (1989). The B allele of the NS gene of avian influenza viruses, but not the A allele, attenuates a human influenza A virus for squirrel monkeys. Virology.

[21] Ludwig S, Schultz U, Mandler J, Fitch WM, Scholtissek C (1991). Phylogenetic relationships of the non-structural (NS) genes of influenza A viruses. Virology.

[22] Long J-X, Peng D-X, Liu Y-L, Wu Y-T, Liu X-F (2008). Virulence of H5N1 avian influenza virus enhanced by a 15-nucleotide deletion in the viral non-structural gene. Virus Genes.

[23] Seo SH, Hoffmann E, Webster RG (2004). The NS1 gene of H5N1 influenza viruses circumvents the host anti-viral cytokine reponse. Virus Res.

[24] Obenauer JC, Denson J, Mehta PK, Su X, Mukatira S, Finkelstein DB, Xu X, Wang J, Ma J, Fan Y, Rakestraw KM, Webster RG, Hoffmann E, Krauss S, Zheng J, Zhang Z, Naeve CW (2006). Large-scale sequence analysis of avian influenza isolates. Science.

[25] Sheng M, Sala C (2001). PDZ domains and the organization of supramolecular] complexes. Annu Rev Neurosci.

[26] Jackson D, Hossain MdJ, Hickman D, Perez D, Lamb RA (2008). A new influenza virus virulence determinant: the NS1 protein four C-terminal residues modulate pathogenicity. Proc Natl Acad Sci USA.

[27] Chen GW, Chang SC, Mok CK, Lo YL, Kung YN, Huang JH, Shih YH, Wang JY, Chiang C, Chen CJ, Shih SR (2006). Genomic signatures of human versus avian influenza viruses. Emerg Infect Dis.

[28] Finkelstein DB, Mukatira S, Mehta PK, Obenauer JC, Su X, Webster RG, Naeve CW (2007). Persistent host markers in pandemic and H5N1 influenza viruses. J Virol.

[29] Monne I, Fusaro A, Al-Blowi MH, Ismail MM, Khan OA, Dauphin G, Tripodi A, Salviato A, Marangon S, Capua I, Cattoli G (2008). Co-circulation of two sublineages of HPAI H5N1 virus in the Kingdom of Saudi Arabia with unique molecular signatures suggesting separate introductions into the commercial poultry and falconry sectors. J Gen Virol.

[30] Heikkinen LS, Kazlauskas A, Melén K, Wagner R, Ziegler T, Julkunen I, Saksela K (2008). Avian and 1918 Spanish influenza A virus NS1 proteins bind to Crk/CrkL Src homology 3 domain to activate host cell signalling. J Biol Chem.

[31] Chen ZLY, Krug RM (1999). Influenza A virus NS1 protein targets poly(A)-binding protein II of the cellular 3′-end processing machinery. EMBO J.

[32] Greenspan D, Palese P, Krystal M (1988). Two nuclear location signals in the influenza virus NS1 nonstructural protein. J Virol.

[33] Melén K, Kinnunen L, Fagerlund R, Ikonen N, Twu KY, Krug RM, Julkunen I (2007). Nuclear and nucleolar targeting of influenza A virus NS1 protein: striking differences between different virus subtypes. J Virol.

[34] Dundon WG, Milani A, Cattoli G, Capua I (2006). Progressive truncation of the Non Structural 1 gene of H7N1 avian influenza viruses following extensive circulation in poultry. Virus Res.

[35] Dundon WG, Rashid S, Mazzacan E, Naeem K, Capua I Sequence analysis of the ns1 gene of H7N3 viruses isolated in Pakistan reveals a 13aa truncation and three viral subpopulations circulating between 1995 and 2005.

[36] Chen H, Smith GJ, Li KS, Wang J, Fan XH, Rayner JM, Vijaykrishna D, Zhang JX, Zhang LJ, Guo CT, Cheung CL, Xu KM, Duan L, Huang K, Qin K, Leung YH, Wu WL, Lu HR, Chen Y, Xia NS, Naipospos TS, Yuen KY, Hassan SS, Bahri S, Nguyen TD, Webster RG, Peiris JS, Guan Y (2006). Establishment of multiple sublineages of H5N1 influenza virus in Asia: implications for pandemic control. Proc Natl Acad Sci USA.

[37] Smith GJ, Fan XH, Wang J, Li KS, Qin K, Zhang JX, Vijaykrishna D, Cheung CL, Huang K, Rayner JM, Peiris JS, Chen H, Webster RG, Guan Y (2006). Emergence and predominance of an H5N1 influenza variant in China. 2006. Proc Natl Acad Sci U S A.

[38] Lindstrom SE, Cox NJ, Klimov A (2004). Genetic analysis of human H2N2 and early H3N2 influenza viruses, 1957–1972: evidence for genetic divergence and multiple reassortment events. Virology.

[39] Govorkova EA, Rehg JE, Krauss S, Yen HL, Guan Y, Peiris M, Nguyen TD, Hanh TH, Puthavathana P, Long HT, Buranathai C, Lim W, Webster RG, Hoffmann E (2005). Lethality to ferrets of H5N1 influenza viruses isolated from humans and poultry in 2004. J Virol.

[40] Salomon R, Franks J, Govorkova EA, Ilyushina NA, Yen HL, Hulse-Post DJ, Humberd J, Trichet M, Rehg JE, Webby RJ, Webster RG, Hoffmann E (2006). The polymerase complex genes contribute to the high virulence of the human H5N1 influenza virus isolate A/Vietnam/1203/04. J Exp Med.

[41] Aamir UB, Wernery U, Ilyushina N, Webster RG (2007). Characterization of avian H9N2 influenza viruses from United Arab Emirates 2000 to 2003. Virol.

[42] Cheung CL, Vijaykrishna D, Smith GJ, Fan XH, Zhang JX, Bahl J, Duan L, Huang K, Tai H, Wang J, Poon LL, Peiris JS, Chen H, Guan Y (2007). J Virol.

[43] Naeem K, Siddique N, Ayaz M, Jalalee MA (2007). Avian influenza in Pakistan: outbreaks of low-and high-pathogenicity avian influenza in Pakistan during 2003–2006. Avian Dis.

[44] Krystal M, Buonagurio D, Young JF, Palese P (1983). Sequential mutations in the NS genes of influenza virus field strains. J Virol.

[45] Norton GP, Tanaka T, Tobita K, Nakada S, Buonaugurio DA, Greespan D, Kyrstal M, Palese P (1987). Infectious influenza A and B virus variants with long carboxyl terminal deletions in the NS1 polypeptides. Virol.

[46] Capua I, Alexander DJ (2008). Avian influenza vaccines and vaccination in birds. Vaccine.

[47] Wang L, Suarez DL, Pantin-Jackwood M, Mibayashi M, Garcìa-Satre A, Saif YM, Lee C-W (2008). Characterization of influenza virus variants with different sizes of the non-structural (NS) genes and their potential as a live influenza vaccine inpoultry. Vaccine.

[48] Zhirnov OP, Isaeva EI, Konakova TE, Thoidis G, Piskareva LM, Akopova II, Kartashov A, Altstein AD, Ilyinskii PO, Shneider AM (2007). Protection against mouse and avian influenza A strains via vaccination with a combination of conserved proteins NP, M1 and NS1. Influenza Other Res. Viruses.

[49] Boon AC, de Mutsert G, Graus YM, Fouchier RA, Sintnicolaas K, Osterhaus AD, Rimmelzwaan GF (2002). The magnitude and specificity of influenza A virus-specific cytotoxic T-lymphocyte responses in humans is related to HLA-A and -B phenotype. J Virol.

[50] Steel J, Lowen AC, Pena L, Angel M, Solórzano A, Albrecht R, Perez DR, García-Sastre A, Palese P (2009). Live attenuated influenza virus containing NS1 trucations as vaccines candidates against H5N1 highly pathogenic avian influenza. J Virol.

[51] Capua I, Terregino C, Cattoli G, Toffan A (2004). Increased resistance of vaccinated turkeys to experimental infection with an H7N3 low-pathogenicity avian influenza virus. Avian Pathol.

[52] Swayne DE, Suarez DL (2000). Highly pathogenic avian influenza. Revue Scientifique Technique Office Internationale des Epizooties.

[53] Capua I, Terregino C, Cattoli G, Mutinelli F, Rodriguez JF (2002). Development of a DIVA (Differentiating Infected from Vaccinated Animals) strategy using a vaccine containing a heterologous neuraminidase for the control of avian influenza. Avian Pathol.

[54] Cattoli G, Terregino C, Brasola V, Rodriguez JF, Capua I (2003). Development and preliminary validation of an ad hoc N1–N3 discriminatory test for the control of avian influenza in Italy. Avian Dis.

[55] Lambrecht B, Steensels M, Van BS, Meulemans G, van den Berg BT (2007). Development of an M2e-specific enzyme-linked immunosorbent assay for differentiating infected from vaccinated animals. Avian Dis.

[56] Kwon JS, Kimm MC, Jeong OM, Kang HM, Song CS, Kwon JH, Lee YJ (2009). Novel use of a N2-specific enzyme-linked immunosorbent assay for differentiation of infected from vaccinated animals (DIVA)-based identification of avian influenza. Vaccine.

[57] Wu R, Chen Q, Zheng L, Chen J, Sui Z, Guan Y, Chen Z (2009). Generation and evaluation of an H9N1 influenza vaccine derived by reverse genetics that allows utilization of a DIVA strategy for control of H9N2 avian influenza. Arch Virol.

[58] Tumpey TM, Alvarez R, Swayne DE, Suarez DL (2005). Diagnostic approach for differentiating infected from vaccinated poultry on the basis of antibodies to NS1, the nonstructural protein of influenza A virus. J Clin Microbiol.

[59] Zhao S, Jin M, Li H, Tan Y, Wang G, Zhang R, Chen H (2005). Detection of antibodies to the nonstructural protein (NS1) of avian influenza viruses allows distinction between vaccinated and infected chickens. Avian Dis.

[60] Birch-Machin I, Rowan A, Pick J, Mumford J, Binns M (1997). Expression of the non-structural protein NS1 of equine influenza A virus: detection of anti-NS1 antibody in post infection equine sea. J Virol Methods.

[61] Ozaki H, Sugiura T, Sugita S, Imagawa H, Kida H (2001). Detection of antibodies to the non-structural protein (NS1) of influenza A virus allows distinction between vaccinated and infected horses. Vet Microbiol.

[62] Dundon WG, Maniero S, Toffan A, Capua I, Cattoli G (2007). Appearance of serum antibodies against the avian influenza non-structural 1 protein in experimentally infected in chickens and turkeys. Avian Dis.

[63] Avellanda G, Mundt E, Lee C-W Differentiation of infected and vaccinated animals (DIVA) using the NS1 protein of avian influenza virus.

